# Introduction to the *JCTS* special issue on Dissemination and Implementation Sciences

**DOI:** 10.1017/cts.2020.488

**Published:** 2020-07-07

**Authors:** Kathleen R. Stevens, Jonathan N. Tobin

**Affiliations:** 1 JCTS Senior Editor, Castella Endowed Distinguished Professor, School of Nursing, University of Texas Health San Antonio, San Antonio, TX, USA; 2 JCTS Co-Deputy Editor, President/CEO, Clinical Directors Network, Inc. (CDN), Professor, Department of Epidemiology and Population Health, Albert Einstein College of Medicine, New York, NY, USA; 3 Co-Director, Community-Engaged Research, Senior Epidemiologist and Adjunct Professor, The Rockefeller University Center for Clinical and Translational Science, New York, NY, USA

## First themed issue for *JCTS*

Together with the Association for Clinical and Translational Science, the Editorial Board of the *Journal for Clinical and Translational Science (JCTS)* has worked over the past two years to raise awareness of the potential of Dissemination and Implementation (D&I) Sciences in improving population health. To that end, this is the first special issue of *JCTS* focused on these emerging scientific fields. This Special Issue has generated significant enthusiasm from those pushing systematic research to this range of the translational science spectrum, where knowledge turns into practice and benefit (hopefully). This collection identifies a body of work that advances translational science. We believe that this themed issue is an excellent vehicle for summarizing a representative selection of leading scientific work stemming from collaborations with (but not limited to) the NIH/NCATS-funded Clinical and Translational Science Award (CTSA) program. We look forward to seeing *JCTS* become a central dissemination partner for D&I Research and scientists going forward.

## Definition of Dissemination, Implementation, and D&I Research

NIH defines implementation as the adoption and integration of evidence-based health interventions into clinical and community settings to improve care delivery and efficiency, patient outcomes, and individual and population health. Implementation Research is the scientific study of methods that promote systematic integration of research findings in routine care and practice. Implementation Science is a specialized field that evaluates strategies to enhance adoption of evidence-based practices into everyday care to improve health and health care. Dissemination Research is the scientific study of active and targeted distribution of information and intervention materials to a specific publich health or clinincial practice audience.^1^ The goal of Dissemination Science is to expand our understanding of effective ways to spread knowledge of evidence-based interventions. Some studies combine both D&I while others focus on specific aspects of either one.^2^

## D&I sciences are rapidly evolving with theories, frameworks, methods, standards, applications and incorporation into studies of comparative effectiveness and public health impact

As with all emerging scientific fields, D&I sciences have been rapidly evolving in the past decade, enjoying a noticeable surge in the last two years. Expansion is occurring in the theories, frameworks, research methods design, analytics, standards of rigor, application to problems, and design of research studies. This *JCTS Special Issue on D&I Research* contains articles spanning many of these topics.

## Broad themes from papers published in this JCTS Special Issue on D&I Research

Several manuscripts in this *JCTS* special issue on D&I Research describe innovative strategies and frameworks designed to enhance and improve the translation of research to practice. Ramsey et al.^3^^*^ presented Design for Accelerated Translation (DART) framework that proposes additional factors, such as demand, risk, and cost all influence the pace of translation of innovations into practice and outlined key actions to accelerate that process. Leppin et al.^4^ propose an integrated framework where D&I sciences are used to identify strategies for routinely and proactively accelerating research translation. Similarly, Hwang et al.^5^ propose utilization of novel implementation science methodologies to advance health in the US and worldwide, while Yousefi et al.^6^ discuss interactions between health equity research and D&I science, underlying the importance of translational research with the focus on equitable D&I. Bennett et al. highlighted a need for research integration into Learning Health Systems (LHS).^7^ Aarons et al.^8^ and Dolor et al.^9^ examined opinions of D&I researchers and CTSA leaders to identify strategies that could promote team science and improve population health. The importance of partnerships between academic and public health systems and organizations is examined by both Kilbourne et al.^10^ and Towfighi et al.^11^ This is further explored in terms of a broader engagement of specific participants, including stakeholder and workforce related issues. Quanbeck et al.^12^ and Meissner et al.^13^ discuss the vital role of stakeholder engagement. Eder at al.^13^ describe practice facilitation as an important implementation strategy to communicate shared project goals and monitor practice behaviors, while Sterling et al.,^15^ examine the role of a specific group of neglected stakeholders – home care workers. Fiscella et al.^16^ discussed the role of data and safety monitoring boards (DSMBs) in implementations trials, raising the question about whether monitoring for differences in effectiveness is necessary or appropriate, and whether additional systems-level implementation outcomes should be monitored, such as impact on workflow and workforce that could create strain on the staff or increase access to care barriers.

The role of past research participants and their perspective on dissemination was described by Melvin et al.^17^ and Cook et al.,^18^ while Mahoney et al.^19^ found that widespread dissemination can be maintained if attention is paid to fidelity of key elements. Mackie et al.^20^ discuss the importance of adaptation as ad-hoc modifications of research protocols and their impact on primary effectiveness and implementation outcomes.

## Conclusion

Taken in their entirety, these exciting reports document some of the key areas for developing scientific consensus in order to advance the field. Science advances by linking observations and measures to theories and mechanisms through reproducible methods and transparent reporting. The gains from D&I Sciences are great through contribution to a wider perspective on how to generate new translational evidence and bring that new evidence to the last mile for the benefit of all. At the same time, such studies should continue to examine, document, and intervene to increase health equity by ensuring that no populations are left out of experiencing the benefits of research. If judged by the quality and diversity of the submissions to this special issue, D&I science is widely deemed an essential part of the CTSA program. D&I Research brings the labors of earlier stages of translational research to full fruition through building effective ways to integrate results into routine clinical care for the general population. As D&I Research sciences continue to evolve, the *JCTS* editors look forward to publishing other noteworthy examples of D&I Research conducted by CTSA teams in the future.

## Post-Script

Since ongoing professional discourse is vital to the evolution of scientific fields and discovery, *JCTS* readers are invited and encouraged to comment on the articles contained in this D&I Special issue through *JCTS* channels, including letters to the editor, and across social media platforms, to keep this lively exchange going.

## Figures and Tables

**Table:** 

Dr. Kathleen R. Stevens, RN, EdD, ANEF, FAAN *JCTS* Senior Editor Castella Endowed Distinguished Professor School of Nursing University of Texas Health San Antonio Collaboration and Engagement Leadership Team, Institute for Integration of Medicine & Science	Jonathan N. Tobin, PhD *JCTS* Co-Deputy Editor President/CEO, Clinical Directors Network, Inc. (CDN) Professor, Department of Epidemiology and Population Health, Albert Einstein College of Medicine Co-Director, Community-Engaged Research, Senior Epidemiologist and Adjunct Professor, The Rockefeller University Center for Clinical and Translational Science, New York NY USA
